# Addressing reproductive health inequities in Brazil's open drug scenes: the case for improving uptake of etonogestrel subdermal implant

**DOI:** 10.61622/rbgo/2025rbgo60

**Published:** 2025-07-15

**Authors:** Clarice Sandi Madruga, Katia Isicawa de Sousa Barreto, Danilo Seabra, André Miguel, Claudio Jerônimo da Silva, Guilherme Sabino de Godoy, Lidiane Nogueira Rebouças, Natália Alexandre Ferreira, Rogerio Adriano Bosso, Carmen Silvia Molleis Galego Miziara, Martha Canfield, Quirino Cordeiro, Ronaldo Ramos Laranjeira

**Affiliations:** 1 Universidade Federal de São Paulo São Paulo SP Brazil Universidade Federal de São Paulo, São Paulo, SP, Brazil.; 2 Associação Paulista para o Desenvolvimento da Medicina São Paulo SP Brazil Associação Paulista para o Desenvolvimento da Medicina, São Paulo, SP, Brazil.; 3 Washington State University Pullman Washington United States Washington State University, Pullman, Washington, United States.; 4 Secretaria da Proteção Social Fortaleza CE Brazil Secretaria da Proteção Social, Fortaleza, CE, Brazil.; 5 Universidade de São Paulo Ribeirão Preto SP Brazil Universidade de São Paulo, Ribeirão Preto, SP, Brazil.; 6 Universidade de São Paulo São Paulo SP Brazil Universidade de São Paulo, São Paulo, SP, Brazil.; 7 Glasgow Caledonian University Glasgow United Kingdom Glasgow Caledonian University, Glasgow, United Kingdom.; 8 Faculdade de Ciências Médicas da Santa Casa de São Paulo São Paulo Brasil Faculdade de Ciências Médicas da Santa Casa de São Paulo, São Paulo, Brasil

Dear Editor,

Brazil has one of the largest crack-cocaine markets in the world.^(1)^ An estimated 6 million Brazilians have used crack-cocaine at least once, and around 1.3 million are considered dependent on the drug.^(2)^ Crack-cocaine dependence poses a significant public health challenge in the country, driven by factors such as easy access, widespread availability, and low cost.^(1)^ Over 98% of Brazilian municipalities report problems related to crack use.^(1)^ As a result, open drug scenes (ODS), which are defined as public spaces where people who use drugs (PWUD) and drug dealers gather for the use and distribution of drugs are a pervasive phenomenon in Brazil's major urban centers.^(3)^ These scenes are considered "open" due to their lack of physical containment, public accessibility, and visibility to bystanders. The issues associated with ODS are often similar across contexts, arising from the intersection of environmental factors, patterns of drug use and distribution, and broader systemic conditions.^(3)^ Despite Brazil's extensive regional and cultural diversity, ODS across the country reflect persistent patterns of social exclusion, rooted in structural inequalities such as poverty, street homelessness, and poor health outcomes.^(3)^ Together, these intersecting issues contribute to the extreme marginalisation of PWUD in ODS, positioning them among one of the most socially excluded populations in the country.

Since 2016, the Survey of Drug Use Scenes in Capitals (LECUCA - *Levantamento de Cenas de Uso em Capitais*) has monitored population size, demographics, health, and substance use patterns in ODS. Six waves of the survey have been undertaken in São Paulo (2016, twice in 2017, 2019, 2022 and 2024), with Brasília and Fortaleza joining in 2022. Findings from LECUCA show that while men are more frequently present in ODS, women tend to have stronger and more prolonged ties to the scenes.^(4)^ Moreover, women in ODS face heightened health and social vulnerabilities, including higher rates of sexually transmitted infections, exposure to physical violence, and a significant prevalence of past and current pregnancies.^(4)^ These findings are in line with previous studies on female crack-users in Brazil, which show that this population is characterised by high-intensity drug consumption, prostitution, pregnancy and vulnerability.^(5)^ In results, crack-cocaine female users in Brazil are at greater risk of HIV, hepatitis B and C, and other sexually acquired infections as they are more likely to engage in high sexual risk behaviours (e.g., unprotected sex) and sex work activities in exchange for money and/or drugs.^(5,6)^

Clinical data suggest that crack use during pregnancy is a major risk factor for maternal morbidity and neonatal complications. Adverse maternal effects associated with crack-cocaine include placental displacement, ruptured uterus, seizures and intracerebral haemorrhage.^(7)^ The most frequently reported neonatal complications associated with prenatal crack-cocaine exposure are prematurity, intrauterine growth restriction, low birth weight and birth defects.^(7)^ The risk of spontaneous abortion and stillbirth is high among crack-cocaine pregnant women.^(7)^ Newborns of crack users may exhibit reduced appetite and diminished reactivity, potentially contributing to malnutrition, with evidence also suggesting an association with altered neurodevelopmental outcomes.^(7)^ It is also clear that crack-cocaine use during pregnancy has physical and mental health implications for newborns beyond the perinatal phase, including poor development and emotional outcomes in adolescence and adulthood.^(8,9)^

For decades, the Brazilian Unified Health System (*SUS – Sistema Único de Saúde*) has been offering a range of free contraceptive methods, including condoms, oral contraceptives, and copper intrauterine devices (IUDs). Despite this, fertility rates among women in ODS remain high. Data from LECUCA 2022^(4)^ showed that among 127 women who use crack-cocaine, 110 (86.6%) reported a lifetime history of pregnancy, with an average of 2.69 (SD = 2.15) live births per woman. In contrast, fertility rates among the general female population in Brazil have declined in recent decades, currently averaging 1.63 births per woman.^(10)^ There is also strong evidence from poor reproductive health outcomes among women who use crack-cocaine.^(7)^ Unintended pregnancies are often reported to be high as well as high rates of adverse pregnancy outcomes (miscarriage, termination and stillbirth).^(7)^ Data from LECUCA 2022 further supports this, with the prevalence of natural/induced termination of 32.29% suggesting that many women in ODS might undertake unsafe methods to end unintended pregnancies.^(4)^

A growing body of evidence indicates that women who use crack-cocaine in Brazil, including those in ODS, often face substantial barriers to accessing healthcare, largely due to lives marked by instability, overlapping social vulnerabilities, and pressing survival needs.^(5)^ In recognition of the unique contraceptive needs of this population (favouring methods that are more reliable than condoms, less maintenance than daily oral contraceptives, and less invasive IUDs), the Brazilian SUS began offering in 2021^(11)^ free access to the etonogestrel subdermal implant, known as Implanon, for women considered ‘vulnerable’ or ‘at social risk’. Implanon is a Long-Acting Reversible Contraception (LARC) inserted under the skin of the inner upper arm by a healthcare provider. It offers up to three years of high contraceptive efficacy (Pearl Index = 0.006)^(12)^ without requiring daily actions and fertility returns promptly after its removal.

Despite the inclusion of Implanon in Brazil, its uptake remains limited among women in ODS. Data from LECUCA 2022^(4)^ indicate that only 14.9% of the surveyed women reported having received the implant. Uptake rates vary significantly by region, with 25.4% in São Paulo, 14.3% in Brasília, and only one case in Fortaleza. Furthermore, overall acceptability of Implanon is low, with many respondents expressing no interest in using the implant: 54.0% in São Paulo, 50.0% in Brasília, and 86.2% in Fortaleza. Notably, a greater proportion of women who had a history of pregnancy were more likely to accept Implanon compared to those who had never been pregnant or who were not using any other form of contraception regularly.^(4)^

The reasons behind women's reluctance to use Implanon in ODS remain unclear. There is an urgent need for qualitative studies to explore these women's perspectives and decision-making processes. What is known, however, is that decisions around LARC are deeply complex and must be situated within broader contexts of healthcare access, reproductive justice, personal autonomy, stigma, and lived experience.^(13,14)^ Incorporating women's understanding of Implanon may improve their uptake, and eliciting this understanding before implementing may ease the incorporation of this clinical service in line into existing practice. In addition, supporting reproductive autonomy for women in ODS requires trust-building, non-coercive counselling and trauma-informed care. Promoting sexual and reproductive rights for women who use drugs also demands close attention to the pervasive impact of gender-based violence on this population. For example, among the women surveyed in LECUCA 2022,^(4)^ 19.44% reported experiencing abuse in childhood and 35.30% had been victims of physical violence within ODS. The intersection of gender, drug use and violence compounds stigma, further underscoring the need for a trauma-informed approach to sexual and reproductive health services.

The differing uptake rates of Implanon across São Paulo, Brasília, and Fortaleza might suggest the presence of region-specific, system-level barriers to the implementation and delivery of contraceptive services within ODS. These barriers may include limited advocacy by local authorities in securing resources, as well as the financial and logistical challenges of educating and training healthcare personnel. São Paulo, home to Brazil's largest and most visible ODS (known as Cracolândia) has long been at the centre of public and political debate surrounding drug use, urban poverty, and public health. In response, several health and social initiatives have been implemented in the area, including targeted reproductive health programs offering Implanon since 2014.^(15)^ Other harm reduction approaches in São Paulo have focused on expanding outreach, offering mobile health services, and integrating reproductive healthcare within broader frameworks addressing substance use, homelessness, and gender-based violence.^(16)^ In contrast, cities like Fortaleza, where uptake of Implanon remains extremely low, may lack comparable initiatives, infrastructure, or political support, further highlighting the uneven landscape of access and service delivery across Brazil.
